# Soda

**Published:** 1878-01

**Authors:** 


### SODA.

J. B. C.

The attention of Congress is about to be called to the importance to this country of some alterations in the tariff, tending to foster efforts, which are now being made, to manufacture soda here in quantities commensurate to its great demand in almost every branch of industry.

The Association of Manufacturing Chemists have, at their late meeting, appointed a committee to memorialize Congress, and already large works are projected should the required concessions be granted.

A few items of importance upon this article and its manufacture may not be uninteresting to the readers of the BISTOURY at this time.

The carbonate of soda is found in an impure native state on the margins of lakes in Egypt, Siberia, Thibet, and on the borders of the Black and Caspian Seas, under the name of "Natrona." It is also extracted from "kryolite," which is found in Iceland in large quantities, and brought to this country by a company who have a monopoly, and manufacture, in Western Pennsylvania, caustic soda and other chemicals from it. But by far the greater quantity of the soda of commerce is manufactured in England from common salt (chloride of sodium). Salt being necessary to life, both to the human and the brute species, is found scattered by bountiful Nature, in various forms, all over the earth, either in a solid crystalline state (rock salt) or as the natural brine of wells or springs, and of the water of the ocean. In whatever form it occurs, however, in order to obtain it pure, evaporation is necessary, either by artificial heat or by exposure, as in the usual process of obtaining salt from sea water, to the rays of the sun, in shallow pools; the salt is precipitated and crystallizes; it is then removed and piled up in sheds, where by the action of the atmosphere the chloride of magnesium is removed—after which it is further refined, if necessary, by being again dissolved and crystallized. Natural brine is obtained in several places in England by boring. In our own country we have numerous salt springs, those of New York and Virginia being the most prominent at this time.

Soda has been manufactured from salt by the "Le Blanc" process in England for more than half a century. This process requires sulphuric acid, and involves the use of an immense amount of fuel in the furnaces in the operation of the decomposition of the salt. It was found impossible in using the common Reverberatory Furnace, as was done for a long time, to keep the hydrochloric acid from escaping into the air; a serious loss to the manufacturer, and involving him in constant law suits with his neighbors on account of the destruction by it of all vegetation within its influence. A special act of Parliament was passed, known as the "Alkali Act," compelling a condensation of this gas not less than 95 per cent. This resulted in the necessary improvements, and the saving of the acid which now enters largely into the manufacture of bleaching powder.

Great Britain has almost a monopoly of this manufacture, and the value of her annual production of soda ash, soda crystals, bicarbonate of soda and bleaching powder is in the neighborhood of three millions pounds sterling—say fifteen millions dollars—giving employment to, in the various branches incident to the manufacture, at least twenty thousand men.

The exports of soda to this country, from Great Britain, for ten months ending October 31st last, amounted to £800,000.

Soda is necessary in the manufacture of glass, paper, soap, textile fabrics, and enters largely into the compounds of artificial fertilizers.

It forms the basis of a number of chemical compounds, many of which are used as medicinal agents.

As stated above, a small (comparatively) quantity of soda is manufactured at present in this country, but works are in progress, or in contemplation, at Cincinnati, at Philadelphia, and at other points, which, with improvements upon the "Le Blanc" method, or entirely new processes, will eventually secure to us this, the most important chemical manufacture of the present day, and one for which we are now almost entirely dependent upon England, and in which there is no reason why we should not have been entirely independent long ago.

Such movements as this are the only true ways out of our present depressed condition, and should have the sympathy of all.

